# RNA-seq data of *Ganoderma boninense* at axenic culture condition and under *in planta* pathogen-oil palm (*Elaeis guineensis* Jacq.) interaction

**DOI:** 10.1186/s13104-019-4652-y

**Published:** 2019-09-24

**Authors:** Mui Yun Wong, Nisha T. Govender, Chia Sui Ong

**Affiliations:** 1 Department of Plant Protection, Faculty of Agriculture, Universiti Putra Malaysia, Serdang, Selangor Malaysia; 2 Institute of Plantation Studies, Universiti Putra Malaysia, Serdang, Selangor Malaysia; 3 Centre for Research in Biotechnology for Agriculture (CEBAR), University Malaya, Kuala Lumpur, Malaysia; 4 Faculty of Information Science and Technology (FIST), Multimedia University, Melaka, Malaysia

**Keywords:** *Ganoderma boninense*, Basidiomycete, Transcriptome, Basal stem rot, Pathogenicity factors

## Abstract

**Objective:**

Basal stem rot disease causes severe economic losses to oil palm production in South-east Asia and little is known on the pathogenicity of the pathogen, the basidiomyceteous *Ganoderma boninense*. Our data presented here aims to identify both the house-keeping and pathogenicity genes of *G. boninense* using Illumina sequencing reads.

**Description:**

The hemibiotroph *G. boninense* establishes via root contact during early stage of colonization and subsequently kills the host tissue as the disease progresses. Information on the pathogenicity factors/genes that causes BSR remain poorly understood. In addition, the molecular expressions corresponding to *G. boninense* growth and pathogenicity are not reported. Here, six transcriptome datasets of *G. boninense* from two contrasting conditions (three biological replicates per condition) are presented. The first datasets, collected from a 7-day-old axenic condition provide an insight onto genes responsible for sustenance, growth and development of *G. boninense* while datasets of the infecting *G. boninense* collected from oil palm-*G. boninense* pathosystem (*in planta* condition) at 1 month post-inoculation offer a comprehensive avenue to understand *G. boninense* pathogenesis and infection especially in regard to molecular mechanisms and pathways. Raw sequences deposited in Sequence Read Archive (SRA) are available at NCBI SRA portal with PRJNA514399, bioproject ID.

## Objective

Basal stem rot (BSR) of oil palm (OP) commences by physical contact of root tissues with the lignin degrading white rot fungi, *G. boninense* that are generally found inhabiting dead and decaying wood debris. The pathogen penetrates via the microhyphae [1] and establishes within host tissue using appressoria [2]. Lignin modifying enzymes (LMEs) are synthesized by white rot fungi to mineralize lignin into carbon dioxide and water [3]. These enzymes are large globular proteins, secreted actively during colonization within the hosts’ niche. During penetration, the LMEs that may have been produced by *G. boninense* are unable to diffuse into host epidermal tissue. At the expense of the host tissue, LMEs are secreted by *G. boninense* to break down the complex wood component into simple nutrients. From a biotrophic nutrition, the pathogen later switches into necrotroph that causes the death of host tissues. As the disease progresses (necrotrophic nutrition), BSR of OPs is manifested with trunk collapses, blacken cortex and stele, drooping of leaves and formation of fruiting bodies [4]. Information on the lignin degrading ability and pathogenicity factors/effectors/genes that causes *G. boninense* infection during BSR of OPs is poorly documented. The RNA-seq data of *G. boninense* obtained from two different conditions (axenic and *in planta*) along with the publically available *G. boninense* genome [5], offer lucrative opportunities to understand the molecular events underpinning growth and BSR disease development; effectors for microhyphae, regulatory network of LME production and pathogenesis factor for penetration and colonization.

## Data description

### *Ganoderma boninense* axenic culture

The *Ganoderma boninense* PER71 culture was obtained from Malaysian Palm Oil Board (MPOB), Malaysia and was maintained on malt extract agar (MEA). From the peripheral region of a 7-day-old primary culture, cubes of 0.5 × 0.5 mm were excised and inoculated onto fresh agar plates (three biological replicates). The Petri dish overturned and placed in a dark chamber was incubated at 25 °C, 16/8 h day/night period. To represent *G. boninense* at axenic condition, the mycelium at 7 days after inoculation were scraped gently from the agar surface and flash frozen in liquid nitrogen for RNA isolation.

### Ganoderma boninense in planta

For plant-pathogen *in planta* condition, rubberwood blocks (RWBs) were used to prepare *Ganoderma boninense* inoculum according to Govender et al. [6]. The 3-month-old oil palm seedlings (*Elaeis guineensis* Jacq. *Dura X Psifera*) were obtained from Sime Darby Sdn. Bhd., Malaysia. The seedlings (three biological replicates) were transplanted into pots (40 × 30 cm) of soil mixture. The planting medium preparation and artificial infection was performed according to Govender et al. [7]. Briefly, each pot received about 2 kg of planting medium. Transplanted seedlings were acclimatized at room temperature for 4 weeks prior to artificial infection with *G. boninense* colonized RWB (RWB-inocula). Each seedling was artificially infected with one RWB-inoculum. Oil palm seedlings were carefully pulled from the soil mixture and the RWB-inoculum was attached below the bole and root tissues were arranged randomly to cover the RWB surface. The RWB-inoculum together with the oil palm seedlings were re-planted into pots of soil mixture. All seedlings were regularly watered and maintained under glasshouse condition. At 1 month post-inoculation, root tissues from the artificially infected oil palm seedlings were collected for RNA isolation.

### RNA isolation

RNA was extracted from 0.1 g (fine powder) samples using the TRIzol method. The quality of the RNA was determined by Agilent 2100 bioanalyzer and only RNA samples fulfilling the minimal requirements (RIN ≥ 6.5, concentration ≥ 20 ng/µL, OD260/280 ≥ 1.8, and OD260/230 ≥ 1.8) were used for library preparation.

### RNA-sequencing

We used Illumina HiSeq1000 platform to sequence the high quality RNA samples obtained from *G. boninense* axenic and *in planta G. boninense*-oil palm (root tissues) interaction. All raw reads obtained were subjected to quality check and a subsequent filtering; sequence reads were (Q > 30) “trimmed” to remove low quality bases using Skewer version 0.1.120 [8]. For *in planta* samples, the good quality reads were aligned against the oil palm reference genome (ASJS00000000.1). Aligned reads were removed to knock out the presence of host RNA using HISAT2 [9]. Next, the filtered reads together with reads obtained from the axenic sample were subjected to de novo assembly using the Trinity pipeline (Trinity version 2.8.4); a set of contiguous sequences (contigs) comprised of full and partial fragments of fungal transcripts were generated [10]. Descriptive information on the *Ganoderma boninense* data sets are presented in Table 1. Samples with a SRA label of *SRS4243090*–*SRS4243092* are *in planta G. boninense and SRS4243093*–*SRS4243095* represent the fungus in an axenic condition.

**Table 1:** Overview of *Ganoderma boninense* data sets available at https://www.ncbi.nlm.nih.gov/bioproject/PRJNA514399

File link	Description of data set	File name	BioSample ID
https://www.ncbi.nlm.nih.gov/sra/SRX5240215	*Axenic Ganoderma boninense*_BR1	SRX5240215	*SAMN10724118*
https://www.ncbi.nlm.nih.gov/sra/SRX5240214	*Axenic Ganoderma boninense_*BR2	SRX5240214	*SAMN10724119*
https://www.ncbi.nlm.nih.gov/sra/SRX5240213	*Axenic Ganoderma boninense* _BR3	SRX5240213	*SAMN10724120*
https://www.ncbi.nlm.nih.gov/sra/SRX5240212	*In planta Ganoderma boninense*_BR1	SRX5240212	*SAMN10724121*
https://www.ncbi.nlm.nih.gov/sra/SRX5240211	*In planta Ganoderma boninense*_BR2	SRX5240211	*SAMN10724122*
https://www.ncbi.nlm.nih.gov/sra/SRX5240210	*In planta Ganoderma boninense_*BR3	SRX5240210	*SAMN10724123*

*BR* biological replicate

### Data

See Table 1.

## Limitations

The transcriptome data of *G. boninense* strain PER71 represents a moderate virulence. There are different strains of *G. boninense* described with variable degree of virulence and any other similar transcriptomic comparisons to *G. boninense* strain PER71 may result to variation at the expression levels of the pathogenicity genes. In addition, other environmental variables may also affect the pathogenicity gene expression; temperature, moisture, osmotic stress, host genotypes and presence/absence of commensal microbes.

## Data Availability

The data presented here are available in the Sequence Read Archive, National Centre for Biotechnology Institute (https://www.ncbi.nlm.nih.gov/bioproject/PRJNA514399).

## Abbreviations

BSR: basal stem rot
OPs: oil palms
LME: lignin modifying enzyme
SRA: Sequence Read Archive
